# Supplementary figures and images for: Assessing the quality of CKD care using process quality indicators: A scoping review
Source: PLoS One. 2024 Dec 10;19(12):e0309973. doi: 10.1371/journal.pone.0309973 (PMC11630614; doi:10.1371/journal.pone.0309973)

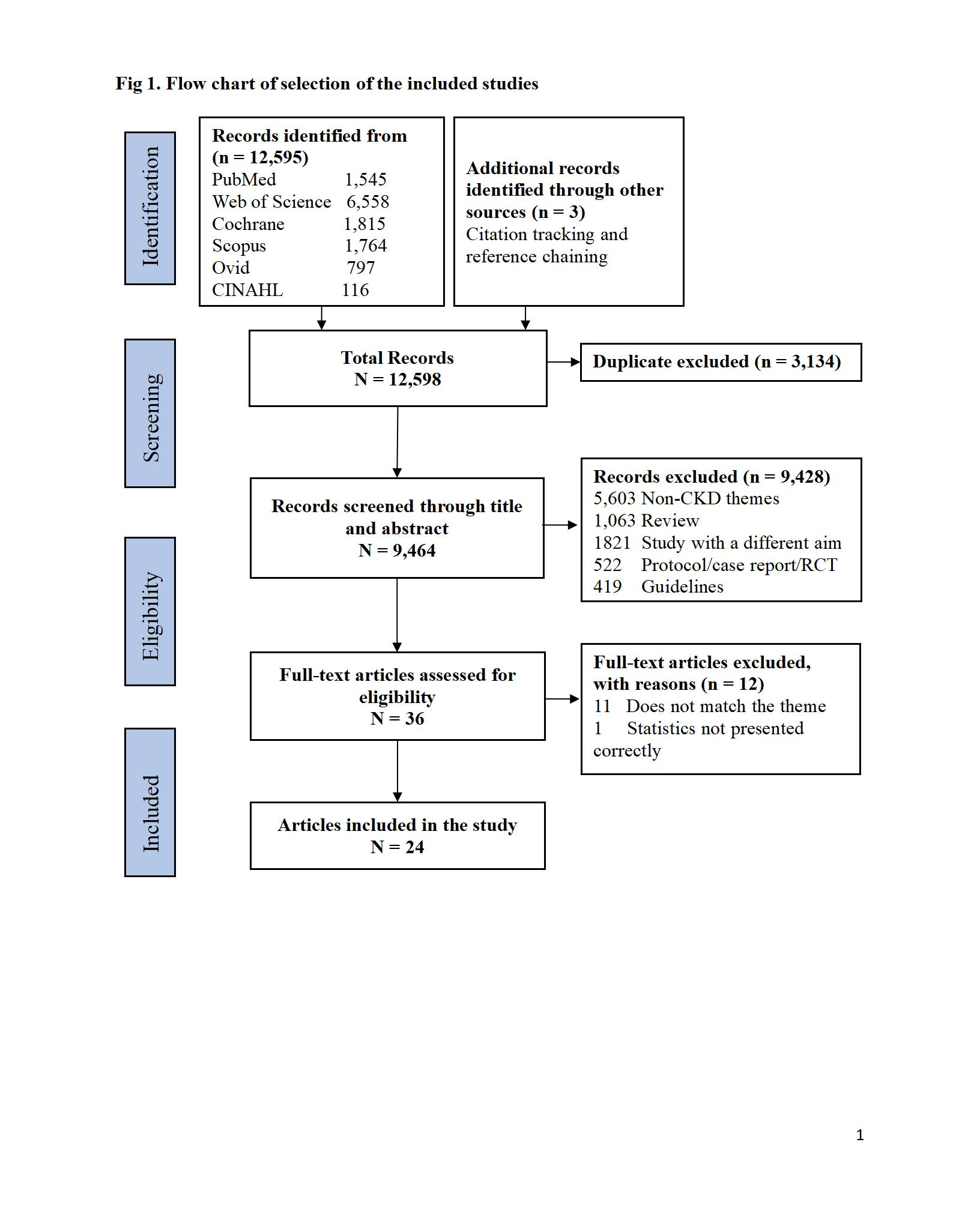

Supplement: S1 Fig — Flow chart of selection of the included studies. (TIF) [file pone.0309973.s001.tif]
