# Supplementary material for: Assessing the quality of CKD care using process quality indicators: A scoping review
Source: PLoS One. 2024 Dec 10;19(12):e0309973. doi: 10.1371/journal.pone.0309973 (PMC11630614; doi:10.1371/journal.pone.0309973)
Supplement: S1 Table — Characteristics of included studies. (DOCX) [file pone.0309973.s002.docx]

# Table 1. Characteristics of included studies

| **Variables** | **N (%)** |
| --- | --- |
| **Study location** |  |
| North America | 10 (41.7) |
| Europe | 8 (33.3) |
| Asia | 4 (16.7) |
| Oceania | 2 (8.3) |
| **CKD eGFR category** |  |
| 3–5 | 12 (50.0) |
| 1–5 | 4 (16.7) |
| 3–4 | 3 (12.5) |
| Unclear | 3 (12.5) |
| 1–4 | 1 (4.2) |
| 3 | 1 (4.2) |
| **Setting** |  |
| Primary care | 15 (62.5) |
| Unspecified/any | 8 (33.3) |
| Secondary care | 1 (4.2) |
| **Study design** |  |
| Cohort study | 17 (70.8) |
| Cross-sectional study | 7 (29.2) |
| **Study population** |  |
| Unspecified/any | 11 (45.8) |
| Population-based | 11 (45.8) |
| Hospital-based | 2 (8.3) |
| **CKD definitions** |  |
| G | 10 (41.7) |
| G/A | 7 (29.2) |
| G/A/Diagnosis code | 6 (25.0) |
| Diagnosis code | 1 (4.2) |
| **Sources of quality indicators** |  |
| National guidelines^a^ | 6 (25.0) |
| Unclear sources | 5 (20.8) |
| Delphi process | 4 (16.7) |
| KDIGO guidelines | 4 (16.7) |
| K/DOQI guidelines | 3 (12.5) |
| Multiple sources^b^ | 2 (8.3) |

Abbreviations: CKD: chronic kidney disease; G: patients were identified as having CKD use estimated glomerular filtration rate (eGFR) criterion for CKD diagnosis; A: patients were identified as having CKD use albuminuria criterion for CKD diagnosis; K/DOQI: Kidney Disease Outcomes Quality Initiative; KDIGO: Kidney Disease Improving Global Outcomes.

^a^ Quality indicators of six studies were derived from national guidelines, including Dutch interdisciplinary CKD-guideline (DIG-CKD) [14, 31], Kidney Health Australia’s CKD guidelines [32, 40], and Clinical Practice Guidelines (CPGs) developed by the National Healthcare Group (NHG) in Singapore [29], and Swedish national guidelines [42].

^b^ Quality indicators of two studies were based on multiple sources: one study was based on recommendations from Canadian Society of Nephrology and previously published data [13], one study used other local guidelines, the 2017 National CKD audit in the United Kingdom, a local audit, and other relevant studies [30].
