# Supplementary material for: Assessing the quality of CKD care using process quality indicators: A scoping review
Source: PLoS One. 2024 Dec 10;19(12):e0309973. doi: 10.1371/journal.pone.0309973 (PMC11630614; doi:10.1371/journal.pone.0309973)
Supplement: S2 Table — Extracted quality indicators and relevant themes. (DOCX) [file pone.0309973.s003.docx]

Table 2. Extracted quality indicators and relevant themes

| **Categories/ Themes/ Quality indicators** | **N of studies (%)** |
| --- | --- |
| **A. Laboratory measures and monitoring of CKD progression and/or complications** | |
| **-Monitoring of kidney markers** | 18 (75.0) |
| -Urine protein | 18 (75.0) |
| -ACR | 9 (37.5) |
| -Any method (unclear) | 4 (16.7) |
| -Urine dipstick protein | 2 (8.3) |
| -PCR | 1 (4.2) |
| -Either ACR or PCR | 1 (4.2) |
| -Scr/eGFR | 14 (58.3) |
| **-CKD-MBD** | 6 (20.8) |
| -Serum calcium | 4 (16.7) |
| -Vitamin D | 3 (12.5) |
| -Phosphate | 2 (8.3) |
| -Parathyroid hormone | 2 (8.3) |
| -Alkaline phosphatase | 1 (4.2) |
| -Calcium-phosphorus product | 1 (4.2) |
| -Serum phosphorus | 1 (4.2) |
| **-Anemia and malnutrition** | 4 (16.7) |
| -Hb | 4 (16.7) |
| -Hb ≥ 10.0 g/dL | 1 (4.2) |
| -Serum albumin | 2 (8.3) |
| -Body Mass Index | 2 (8.3) |
| -Nutritional guidance | 1 (4.2) |
| **-Electrolytes** | 4 (16.7) |
| -Serum potassium | 4 (16.7) |
| **-Volume** | 1 (4.2) |
| -Adequate volume control | 1 (4.2) |
| **B. Use of guideline-recommended therapeutic agents** | |
| **-** **Use of medications** | 22 (91.7) |
| -ACEIs/ARBs | 20 (83.3) |
| -Statins | 16 (66.7) |
| - Avoidance of NSAIDs | 11 (45.8) |
| -Sodium-glucose Cotransporter-2 Inhibitors | 1 (4.2) |
| **C. Attainment of therapeutic targets** | |
| **-Management of blood pressure** | 14 (58.3) |
| -<130/80 mmHg | 12 (50.0) |
| -<140/90 mmHg | 10 (41.7) |
| -No specific target | 8 (33.3) |
| -<135/85 mmHg | 1 (4.2) |
| **-Glycemia** | 9 (37.5) |
| -HbA1c | 5 (20.8) |
| -HbA1c ≤7.0% | 3 (12.5) |
| -Fasting glucose | 4 (16.7) |
| **-Lipids** | 5 (20.8) |
| -LDL-C | 2 (8.3) |
| -LDL | 1 (4.2) |
| -Total cholesterol | 1 (4.2) |

Abbreviations: ACR: albumin-to-creatinine ratio; PCR: protein-to-creatinine ratio; Scr: serum creatinine; eGFR: estimated glomerular filtration rate; CKD-MBD: Chronic Kidney Disease-Mineral and Bone Disorder; Hb: hemoglobin; ACEIs: angiotensin-converting enzyme inhibitors; ARBs: angiotensin receptor blockers; NSAIDs: non-steroidal anti-inflammatory drugs; HbA1c: glycated hemoglobin; LDL-C: low-density lipoprotein cholesterol.
