# Supplementary material for: Assessing the quality of CKD care using process quality indicators: A scoping review
Source: PLoS One. 2024 Dec 10;19(12):e0309973. doi: 10.1371/journal.pone.0309973 (PMC11630614; doi:10.1371/journal.pone.0309973)
Supplement: S3 Table — Proportion of people with CKD meeting selected quality indicators in included studies. (DOCX) [file pone.0309973.s004.docx]

Table 3. Proportion of people with CKD meeting selected quality indicators in included studies

| **Study, year** | **A-Kidney markers** | |  | **B-Use of medications** | | |  | **C-Blood pressure (mmHg)/Glycemia** | | | | |
| --- | --- | --- | --- | --- | --- | --- | --- | --- | --- | --- | --- | --- |
|  | Urine protein | Scr/eGFR |  | ACEIs/  ARBs | Statins | Avoidance of NSAIDs |  | <130/80 | <140/90 | No target | HbA1c |  |
|  |  |  |  |  |  |  |  |  |  |  |  |  |
| Agvall 2023 |  | 65 |  |  |  |  |  |  |  | 79 |  |  |
| Allen 2011 | 30 | 86 |  | 75.0^a^ | 42.0^a^ | 90 |  | 54.0 ^a^ |  |  |  |  |
| Ang 2013 |  |  |  | 88.2^c^ | 87.1^d^ |  |  | 36.3 |  |  |  |  |
| Bansal 2020 | NA | NA |  | 41 | 41 | 69 |  | 40 | 36 |  |  |  |
| Bello 2019 | 27 | 85.5 |  | 36.7^b,c^ | 36.7 |  |  | 59.6^b,c^ | 81.4 | 75.7 | 85.9^c^ |  |
| Bezabhe 2020 |  |  |  | 69.9 | 40.8 | 85.7 |  |  |  |  |  |  |
| Burdge 2019 | 20.8 | 80.5 |  | 42.9 |  |  |  |  |  | 71.8 |  |  |
| Fukuma 2020 | 59.9 |  |  |  |  | 91.2 |  |  |  |  |  |  |
| Jäger 2022 | 18.1 | 70 |  | 70.7^f^ | 49.8 | 82.6 |  | 54 | 54.9 |  |  |  |
| Jamaluddin 2021 | 94.3 |  |  | 81.4 | 92.2 | 89.3 |  | 37 | 66.7 | 45.3 | 98.5^c^ |  |
| Karen 2017 | 34.2 | 90.4 |  | 74.7^b,c^ | 60.4 | 98.6 |  | 65.2 ^c^ |  | 86.1^b,c^ |  |  |
| Khanam 2019 | 19.7 | 89.6 |  |  |  |  |  |  |  | 91.3 |  |  |
| Leszek 2015 |  |  |  | 74 |  |  |  |  |  |  |  |  |
| Luk 2016 |  |  |  | 49 | 53.6 |  |  | 20.8 |  |  |  |  |
| Manns 2017 | 82.6 | 73.2 |  | 58.1 | 39.2 |  |  |  |  |  |  |  |
| Nash 2017 | 70 | 91 |  | 75 | 65 | 84 |  |  |  |  |  |  |
| Rosenthal 2006 |  |  |  | 59.3 |  |  |  | 39 |  |  | 63.0^c^ |  |
| Rucker 2011 | NA |  |  | NA | NA |  |  |  |  |  | NA |  |
| Samal 2014 | 40 | 94 |  | 65 |  |  |  | 45 | 71 |  |  |  |
| Smits 2019 |  |  |  | 56.5 | 41.8 | 99.1 |  |  |  |  |  |  |
| Swartling 2022 | NA | NA |  | NA | NA |  |  |  |  |  |  |  |
| van Dipten 2017 | 40.7 | 71.2 |  |  |  |  |  | 20.1 | 51.2 | 64.5 |  |  |
| Van Gelder 2016 | 47.8 | 82.1 |  | 55.9 | 47 | 78.7 |  | 16.4 | 43.1 | 71.9 |  |  |
| Yuen 2023 | 45.7 |  |  | 54.5 | 39.21 | 94.75 |  |  | 61.96 | 90.21 | 93.0^c^ |  |
| No. of eligible studies | 14 | 12 |  | 18 | 14 | 11 |  | 12 | 8 | 9 | 4 |  |
| Median | 40.4 | 83.8 |  | 62.2 | 44.5 | 89.3 |  | 39.5 | 58.4 | 75.7 | 89.5 |  |
| Interquartile range | 25.5, 62.4 | 77.7, 90.2 |  | 53.1, 74.8 | 40.4, 61.6 | 82.6, 94.8 |  | 24.7, 54.0 | 45.1, 69.9 | 68.2, 88.2 | 68.7, 97.1 |  |

Abbreviations: A. Laboratory measures and monitoring of CKD progression and/or complications; B. Use of guideline-recommended therapeutic agents; C. Attainment of therapeutic targets; Scr: serum creatinine; eGFR: estimated glomerular filtration rate; ACEIs: angiotensin-converting enzyme inhibitors; ARBs: angiotensin receptor blockers; NSAIDs: non-steroidal anti-inflammatory drugs; HbA1c: glycated haemoglobin.

Data were presented for whole study sample, otherwise for subgroups of ^a^ cardiovascular disease, ^b^ Proteinuria, ^c^ Diabetes Mellitus, ^d^ Dyslipidemia, ^e^ hypertension, ^f^ CKD G1– G4.

NA: Quality indicators were examined but data were not available.
