# Supplementary material for: Assessing the quality of CKD care using process quality indicators: A scoping review
Source: PLoS One. 2024 Dec 10;19(12):e0309973. doi: 10.1371/journal.pone.0309973 (PMC11630614; doi:10.1371/journal.pone.0309973)
Supplement: S4 Table — Proportion of people with CKD meeting quality indicators in included studies, by comorbidity status. (DOCX) [file pone.0309973.s005.docx]

Table 4. Proportion of people with CKD meeting quality indicators in included studies, by comorbidity status

| **Study, year** | **A-Kidney markers** | |  | **B-Use of medications** | | |  | **C-Blood pressure (mmHg)/Glycemia** | | | |
| --- | --- | --- | --- | --- | --- | --- | --- | --- | --- | --- | --- |
|  | Urine protein | Scr/eGFR |  | ACEIs/ARBs | Statins | Avoidance of NSAIDs |  | <130/80 | <140/90 | No target | HbA1c |
|  |  |  |  |  |  |  |  |  |  |  |  |
| **Ang 2013^a^** |  |  |  |  |  |  |  |  |  |  |  |
| DM- HT- |  |  |  | 27.9 | NA |  |  | NA |  |  | NA |
| DM + |  |  |  | 88.2 |  |  |  |  |  |  | 39.4 |
| HT + |  |  |  | 84.1 |  |  |  | 36.3/75 |  |  |  |
| **Bello 2019^b^** |  |  |  |  |  |  |  |  |  |  |  |
| DM - / HT - | 9.9 | 77.3 |  | 27.3 | 20.3 |  |  | 53.2 | 89.2 | 65.7 | NA |
| DM & HT + | 48.7 | 91.2 |  | 30.5 | 50.3 |  |  |  | 79.3 | 79.1 | 86 |
| DM + | 45.5 | 89.2 |  | 18.9 | 42.9 |  |  | 57.3 | 86.8 | 75.6 | 85.7 |
| HT + | 16.1 | 84.5 |  | 54.9 | 33.4 |  |  | 67.8 | 78.9 | 77.2 | NA |
| **Fukuma 2020** |  |  |  |  |  |  |  |  |  |  |  |
| DM - | 55.6 |  |  |  |  | 90.5 |  |  |  |  |  |
| DM + | 71.4 |  |  |  |  | 93.3 |  |  |  |  |  |
| **Khanam 2019^c^** |  |  |  |  |  |  |  |  |  |  |  |
| DM - | 19.7^c^ | 89.6 |  |  |  |  |  |  |  | 91.3 |  |
| DM + | 68.7 ^c^ | 95.4 |  |  |  |  |  |  |  | 94.1 |  |
| **Leszek 2015^d^** |  |  |  |  |  |  |  |  |  |  |  |
| CV/DM - |  |  |  | 70.1 |  |  |  |  |  |  |  |
| CV/DM + |  |  |  | 81.4 |  |  |  |  |  |  |  |
| Proteinuria - |  |  |  | 72.4 |  |  |  |  |  |  |  |
| Proteinuria + |  |  |  | 77.2 |  |  |  |  |  |  |  |
| **Manns 2017** |  |  |  |  |  |  |  |  |  |  |  |
| DM - | 78.9 |  |  | 58.1 | 39.2 |  |  |  |  |  |  |
| DM + | 89.9 |  |  | 78.3 | 64.6 |  |  |  |  |  |  |

Abbreviations: A. Laboratory measures and monitoring of CKD progression and/or complications; B. Use of guideline-recommended therapeutic agents; C. Attainment of therapeutic targets; Scr: serum creatinine; eGFR: estimated glomerular filtration rate; ACEIs: angiotensin-converting enzyme inhibitors; ACEIs: angiotensin-converting enzyme inhibitors; ARBs: angiotensin receptor blockers; NSAIDs: non-steroidal anti-inflammatory drugs; HbA1c: glycated haemoglobin.

DM: Diabetes Mellitus; HT: hypertension; CV: cardiovascular disease.

+/- indicates comorbid condition present or absent.

^a^ The study by Ang 2013 reported data for multiple years; values in this table were based on the year 2011.

^b^ The study by Bello 2019 reported data for HbA1c for the 0-1 year and 1-2 year time periods; values in this table were based on the 0-1 year time period.

^c^ The study by Khanam 2019 used ACR to measure urine protein.

^d^The study by Leszek 2015 reported data for multiple years; values in this table were based on the year 2011.
