# Supplementary material for: Assessing the quality of CKD care using process quality indicators: A scoping review
Source: PLoS One. 2024 Dec 10;19(12):e0309973. doi: 10.1371/journal.pone.0309973 (PMC11630614; doi:10.1371/journal.pone.0309973)
Supplement: S2 File — (DOCX) [file pone.0309973.s007.docx]

# Search strategy

Ovid (Medline)

Searched on June 20, 2024

| **Search** | **Query** | |
| --- | --- | --- |
| #1 | (Renal Insufficiency, Chronic or Chronic Renal Insufficiencies or Renal Insufficiencies, Chronic or Chronic Renal Insufficiency or Kidney Insufficiency, Chronic or Chronic Kidney Insufficiency or Chronic Kidney Insufficiencies or Kidney Insufficiencies, Chronic or Chronic Kidney Diseases or Chronic Kidney Disease or Disease, Chronic Kidney or Diseases, Chronic Kidney or Kidney Disease, Chronic or Kidney Diseases, Chronic or Chronic Renal Diseases or Chronic Renal Disease or Disease, Chronic Renal or Diseases, Chronic Renal or Renal Disease, Chronic or Renal Diseases, Chronic).ti,ab,kw. | |
| #2 | (Quality Indicators, Health Care or Quality Indicators, Healthcare or Healthcare Quality Indicator or Healthcare Quality Indicators or Indicator, Healthcare Quality or Indicators, Healthcare Quality or Quality Indicator, Healthcare or Health Metrics or Health Metric or Metrics, Health or Health Care Quality or Quality Indicators or Quality Improvement or Quality of Care or Care Quality or Quality of Health Care or Quality of Healthcare or Healthcare Quality).ti,ab,kw. | |
| #3 | #1 AND #2 | 797 |

PubMed

Searched on June 20, 2024

| **Search** | **Query** | |
| --- | --- | --- |
| #1 | (((((((((((((((((((((Chronic Renal Insufficiencies[Title/Abstract])) OR (Renal Insufficiencies, Chronic[Title/Abstract])) OR (Chronic Renal Insufficiency[Title/Abstract])) OR (Kidney Insufficiency, Chronic[Title/Abstract])) OR (Chronic Kidney Insufficiency[Title/Abstract])) OR (Chronic Kidney Insufficiencies[Title/Abstract])) OR (Kidney Insufficiencies, Chronic[Title/Abstract])) OR (Chronic Kidney Diseases[Title/Abstract])) OR (Chronic Kidney Disease[Title/Abstract])) OR (Disease, Chronic Kidney[Title/Abstract])) OR (Diseases, Chronic Kidney[Title/Abstract])) OR (Kidney Disease, Chronic[Title/Abstract])) OR (Kidney Diseases, Chronic[Title/Abstract])) OR (Chronic Renal Diseases[Title/Abstract])) OR (Chronic Renal Disease[Title/Abstract])) OR (Disease, Chronic Renal[Title/Abstract])) OR (Diseases, Chronic Renal[Title/Abstract])) OR (Renal Disease, Chronic[Title/Abstract])) OR (Renal Diseases, Chronic[Title/Abstract]))) OR ("Renal Insufficiency, Chronic"[Mesh]) | |
| #2 | (((((((((((((((((((Quality Indicators, Healthcare[Title/Abstract])) OR (Healthcare Quality Indicator[Title/Abstract])) OR (Healthcare Quality Indicators[Title/Abstract])) OR (Indicator, Healthcare Quality[Title/Abstract])) OR (Indicators, Healthcare Quality[Title/Abstract])) OR (Quality Indicator, Healthcare[Title/Abstract])) OR (Health Metrics[Title/Abstract])) OR (Health Metric[Title/Abstract])) OR (Metrics, Health[Title/Abstract])) OR (Health Care Quality[Title/Abstract])) OR (Quality Indicators[Title/Abstract])) OR (Quality Improvement[Title/Abstract])) OR (Quality of Care[Title/Abstract])) OR (Care Quality[Title/Abstract])) OR (Quality of Health Care[Title/Abstract])) OR (Quality of Healthcare[Title/Abstract])) OR (Healthcare Quality[Title/Abstract]))) OR ("Quality Indicators, Health Care"[Mesh]) | |
| #3 | #1 AND #2 | 1545 |

Cochrane Library

Searched on June 20, 2024

| **Search** | **Query** | |
| --- | --- | --- |
| #1 | (Chronic Renal Insufficiencies):ab,ti,kw OR (Renal Insufficiencies, Chronic):ab,ti,kw OR (Chronic Renal Insufficiency):ab,ti,kw OR (Kidney Insufficiency, Chronic):ab,ti,kw OR (Chronic Kidney Insufficiency):ab,ti,kw OR (Chronic Kidney Insufficiencies):ab,ti,kw OR (Kidney Insufficiencies, Chronic):ab,ti,kw OR (Chronic Kidney Diseases):ab,ti,kw OR (Chronic Kidney Disease):ab,ti,kw OR (Disease, Chronic Kidney):ab,ti,kw OR (Diseases, Chronic Kidney):ab,ti,kw OR (Kidney Disease, Chronic):ab,ti,kw OR (Kidney Diseases, Chronic):ab,ti,kw OR (Chronic Renal Diseases):ab,ti,kw OR (Chronic Renal Disease):ab,ti,kw OR (Disease, Chronic Renal):ab,ti,kw OR (Diseases, Chronic Renal):ab,ti,kw OR (Renal Disease, Chronic):ab,ti,kw OR (Renal Diseases, Chronic):ab,ti,kw | |
| #2 | (Quality Indicators, Healthcare):ab,ti,kw OR (Healthcare Quality Indicator):ab,ti,kw OR (Healthcare Quality Indicators):ab,ti,kw OR (Indicator, Healthcare Quality):ab,ti,kw OR (Indicators, Healthcare Quality):ab,ti,kw OR (Quality Indicator, Healthcare):ab,ti,kw OR (Health Metrics):ab,ti,kw OR (Health Metric):ab,ti,kw OR (Metrics, Health):ab,ti,kw OR (Health Care Quality):ab,ti,kw OR (Quality Indicators):ab,ti,kw OR (Quality Improvement):ab,ti,kw OR (Quality of Care):ab,ti,kw OR (Care Quality):ab,ti,kw OR (Quality of Health Care):ab,ti,kw OR (Quality of Healthcare):ab,ti,kw OR (Healthcare Quality):ab,ti,kw | |
| #3 | #1 AND #2 | 1815 |

Web of Science (Core Collection)

Searched on June 20, 2024

| **Search** | **Query** | |
| --- | --- | --- |
| #1 | TS = (Renal Insufficiency, Chronic OR Chronic Renal Insufficiencies OR Renal Insufficiencies, Chronic OR Chronic Renal Insufficiency OR Kidney Insufficiency, Chronic OR Chronic Kidney Insufficiency OR Chronic Kidney Insufficiencies OR Kidney Insufficiencies, Chronic OR Chronic Kidney Diseases OR Chronic Kidney Disease OR Disease, Chronic Kidney OR Diseases, Chronic Kidney OR Kidney Disease, Chronic OR Kidney Diseases, Chronic OR Chronic Renal Diseases OR Chronic Renal Disease OR Disease, Chronic Renal OR Diseases, Chronic Renal OR Renal Diseases, Chronic) | |
| #2 | TS = (Quality Indicators, Health Care OR Quality Indicators, Healthcare OR Healthcare Quality Indicator OR Healthcare Quality Indicators OR Indicator, Healthcare Quality OR Indicators, Healthcare Quality OR Quality Indicator, Healthcare OR Health Metrics OR Health Metric OR Metrics, Health OR Health Care Quality OR Quality Indicators OR Quality Improvement OR Quality of Care OR Care Quality OR Quality of Health Care OR Quality of Healthcare OR Healthcare Quality) | |
| #3 | #1 AND #2 | 6558 |

Scopus

Searched on June 20, 2024

| **Search** | **Query** | |
| --- | --- | --- |
| #1 | "Renal Insufficiency, Chronic" OR "Chronic Renal Insufficiencies" OR "Renal Insufficiencies, Chronic" OR "Chronic Renal Insufficiency" OR "Kidney Insufficiency, Chronic" OR "Chronic Kidney Insufficiency" OR "Chronic Kidney Insufficiencies" OR "Kidney Insufficiencies, Chronic" OR "Chronic Kidney Diseases" OR "Chronic Kidney Disease" OR "Disease, Chronic Kidney" OR "Diseases, Chronic Kidney" OR "Kidney Disease, Chronic" OR "Kidney Diseases, Chronic" OR "Chronic Renal Diseases" OR "Chronic Renal Disease" OR "Disease, Chronic Renal" OR "Diseases, Chronic Renal" OR "Renal Disease, Chronic" OR "Renal Diseases, Chronic" | |
| #2 | "Quality Indicators, Health Care" OR "Quality Indicators, Healthcare" OR "Healthcare Quality Indicator" OR "Healthcare Quality Indicators" OR "Indicator, Healthcare Quality" OR "Indicators, Healthcare Quality" OR "Quality Indicator, Healthcare" OR "Health Metrics" OR "Health Metric" OR "Metrics, Health" OR "Health Care Quality" OR "Quality Indicators" OR "Quality Improvement" OR "Quality of Care" OR "Care Quality" OR "Quality of Health Care" OR "Quality of Healthcare" OR "Healthcare Quality" | |
| #3 | #1 AND #2 | 1764 |

CINAHL

Searched on June 20, 2024

| **Search** | **Query** | |
| --- | --- | --- |
| #1 | Renal Insufficiency, Chronic or Chronic Renal Insufficiencies or Renal Insufficiencies, Chronic or Chronic Renal Insufficiency or Kidney Insufficiency, Chronic or Chronic Kidney Insufficiency or Chronic Kidney Insufficiencies or Kidney Insufficiencies, Chronic or Chronic Kidney Diseases or Chronic Kidney Disease or Disease, Chronic Kidney or Diseases, Chronic Kidney or Kidney Disease, Chronic or Kidney Diseases, Chronic or Chronic Renal Diseases or Chronic Renal Disease or Disease, Chronic Renal or Diseases, Chronic Renal or Renal Disease, Chronic or Renal Diseases, Chronic | |
| #2 | Quality Indicators, Health Care or Quality Indicators, Healthcare or Healthcare Quality Indicator or Healthcare Quality Indicators or Indicator, Healthcare Quality or Indicators, Healthcare Quality or Quality Indicator, Healthcare or Health Metrics or Health Metric or Metrics, Health or Health Care Quality or Quality Indicators or Quality Improvement or Quality of Care or Care Quality or Quality of Health Care or Quality of Healthcare or Healthcare Quality | |
| #3 | SU ( Renal Insufficiency, Chronic or Chronic Renal Insufficiencies or Renal Insufficiencies, Chronic or Chronic Renal Insufficiency or Kidney Insufficiency, Chronic or Chronic Kidney Insufficiency or Chronic Kidney Insufficiencies or Kidney Insufficiencies, Chronic or Chronic Kidney Diseases or Chronic Kidney Disease or Disease, Chronic Kidney or Diseases, Chronic Kidney or Kidney Disease, Chronic or Kidney Diseases, Chronic or Chronic Renal Diseases or Chronic Renal Disease or Disease, Chronic Renal or Diseases, Chronic Renal or Renal Disease, Chronic or Renal Diseases, Chronic or ) AND SU ( Quality Indicators, Health Care or Quality Indicators, Healthcare or Healthcare Quality Indicator or Healthcare Quality Indicators or Indicator, Healthcare Quality or Indicators, Healthcare Quality or Quality Indicator, Healthcare or Health Metrics or Health Metric or Metrics, Health or Health Care Quality or Quality Indicators or Quality Improvement or Quality of Care or Care Quality or Quality of Health Care or Quality of Healthcare or Healthcare Quality ) | 116 |
