# Supplementary material for: Assessing the quality of CKD care using process quality indicators: A scoping review
Source: PLoS One. 2024 Dec 10;19(12):e0309973. doi: 10.1371/journal.pone.0309973 (PMC11630614; doi:10.1371/journal.pone.0309973)
Supplement: S3 File — (DOCX) [file pone.0309973.s008.docx]

Characteristics of included studies

| Author, Year, Country | Study design | Setting | CKD eGFR category | Sample size | Sources of  quality indicators | Study population | Themes | Quality indicators (%) |
| --- | --- | --- | --- | --- | --- | --- | --- | --- |
| Agvall 2023,  Sweden | Observational study | Unspecified/any | G1– G5 | 20,488 | National guidelines^a^ | Population-based | **No. of themes: 3** | **No. of quality indicators: 3** |
|  |  |  |  |  |  |  | Monitoring of kidney markers | eGFR (65.0) |
|  |  |  |  |  |  |  | Management of BP | No specific target (79.0) |
|  |  |  |  |  |  |  | Glycemia | Glucose test (76.0) |
| Allen 2011, America | Retrospective cohort study | Primary care | G3 or G4 | 11,774 | K/DOQI | Population-based | **No. of themes: 6** | **No. of quality indicators: 12** |
|  |  |  |  |  |  |  | Monitoring of kidney markers | Urine protein (30.0); |
|  |  |  |  |  |  |  |  | eGFR (86.0) |
|  |  |  |  |  |  |  | CKD- MBD | Serum calcium (45.0); |
|  |  |  |  |  |  |  |  | Parathyroid hormone (13.0) |
|  |  |  |  |  |  |  |  | Vitamin D (24.0) |
|  |  |  |  |  |  |  | Anemia and malnutrition | Hb (76.0) |
|  |  |  |  |  |  |  | Use of medications | ACEIs/ARBs (CV 75.0); |
|  |  |  |  |  |  |  |  | Statin (CV 42.0); |
|  |  |  |  |  |  |  |  | NSAIDs (10.0) |
|  |  |  |  |  |  |  | Management of BP | < 130/80 mmHg (CV 54.0) |
|  |  |  |  |  |  |  | Lipids | LDL-C (CV 74.0); |
|  |  |  |  |  |  |  |  | LDL-C < 2.6 mmol/L (CV 44.0) |
| Ang 2013^b^, Singapore | Retrospective study | Unspecified/any | G3– G5 | 10,245 | National guidelines^a^ | Population-based | **No. of themes: 4** | **No. of quality indicators: 5** |
|  |  |  |  |  |  |  | Use of medications | ACEIs and/or ARBs: |
|  |  |  |  |  |  |  |  | with Hypertension: 84.1 |
|  |  |  |  |  |  |  |  | with DM: 88.2 |
|  |  |  |  |  |  |  |  | Statin (with Dyslipidemia 87.1) |
|  |  |  |  |  |  |  | Management of BP | < 130/? mmHg (36.3) |
|  |  |  |  |  |  |  |  | < ?/80 mmHg (75.0) |
|  |  |  |  |  |  |  | Glycemia | HbA1c ≤ 7.0% (with DM: 39.4) |
|  |  |  |  |  |  |  | Lipids | LDL-C < 2.6 mmol/L (with dyslipidemia: 54.7) |
| Bansal 2020, America | Retrospective cohort study | Primary care | G1- G5 | 92,900 | Unclear sources | Unspecified/any | **No. of themes: 3** | **No. of quality indicators: 7** |
|  |  |  |  |  |  |  | Monitoring of kidney markers | ACR; |
|  |  |  |  |  |  |  |  | Scr/eGFR |
|  |  |  |  |  |  |  | Use of medications | ACEIs/ARBs (41); |
|  |  |  |  |  |  |  |  | Statin (41); |
|  |  |  |  |  |  |  |  | NSAIDs (31) |
|  |  |  |  |  |  |  | Management of BP | < 140/90 mmHg (36); |
|  |  |  |  |  |  |  |  | < 130/80 mmHg (40) |
| Bello 2019, Canada | Cross-sectional study | Primary care | G3- G5 | 46,162 | Multiple sources^c^ | Population-based | **No. of themes: 4** | **No. of quality indicators: 8** |
|  |  |  |  |  |  |  | Monitoring of kidney markers | ACR (27.0); |
|  |  |  |  |  |  |  | (18 mo after confirm CKD) | Scr (85.5) |
|  |  |  |  |  |  |  | Use of medications | ACEIs/ARBs (with proteinuria and/or DM: 36.7); |
|  |  |  |  |  |  |  | (1y after confirm CKD) | Statin (36.7) |
|  |  |  |  |  |  |  |  | Monitoring of ACEI and ARB (within 7 to 30 days): Scr (26.7) |
|  |  |  |  |  |  |  | Management of BP | No specific target (75.7); |
|  |  |  |  |  |  |  |  | ≤140/90 mmHg (81.4); |
|  |  |  |  |  |  |  |  | ≤130/80 mmHg (proteinuria and/or DM: 59.6) |
|  |  |  |  |  |  |  | Glycemia | HbA1c: |
|  |  |  |  |  |  |  | (with DM within first1-2 years) | 0-1 y (85.9); |
|  |  |  |  |  |  |  |  | 1-2 y (66.9) |
| Bezabhe, 2020, Australian | Cross-sectional study | Primary care | G3– G5 | 44,259 | National guidelines^a^ | Population-based | **No. of themes: 2** | **No. of quality indicators: 7** |
|  |  |  |  |  |  |  | CKD- MBD | Vitamin D: (aged≥ 18 with G3– G5 and elevated calcium levels (5.0) |
|  |  |  |  |  |  |  | Use of medications | ACEIs/ARBs: |
|  |  |  |  |  |  |  |  | Microalbuminuria: (69.9); |
|  |  |  |  |  |  |  |  | DM with macroalbuminuria: (62.3) |
|  |  |  |  |  |  |  |  | ACEIs/ARBs plus a diuretic: Microalbuminuria and DM (20.6); Macroalbuminuria (20.4) |
|  |  |  |  |  |  |  |  | Statin: (aged between 50 and 65 years (40.8) |
|  |  |  |  |  |  |  |  | NSAIDs (14.3) |
|  |  |  |  |  |  |  |  | Metformin (14.1) |
|  |  |  |  |  |  |  |  | Phosphate binder (6.6) |
| Burdge, 2019, Ameriaca | Cohort study |  | G3– G5 | 60,503 | KIDIGO | Population-based | **No. of themes: 3** | **No. of quality indicators: 4** |
|  |  |  |  |  |  |  | Monitoring of kidney markers | Urine protein (20.8); |
|  |  |  |  |  |  |  |  | Scr/eGFR (80.5) |
|  |  |  |  |  |  |  | Use of medications | ACEIs/ARBs: (42.9) |
|  |  |  |  |  |  |  | Management of BP | No specific target (71.8) |
| Fukuma 2020, Japan | Cohort study | Unspecified/any | unclear | 890,773 | Delphi process | Population-based | **No. of themes:3** | **No. of quality indicators: 3** |
|  |  |  |  |  |  |  | Monitoring of kidney markers | Urine protein (59.9) |
|  |  |  |  |  |  |  | Anemia and malnutrition | Nutritional guidance (4.5) |
|  |  |  |  |  |  |  | Use of medications | Avoidance of NSAIDs (91.2)) |
| Jäger 2022, Swiss | Retrospective cohort study | General practice | G1– G4 | 14,627 | KDIGO | Population-based | **No. of themes: 4** | **No. of quality indicators: 8** |
|  |  |  |  |  |  |  | Monitoring of kidney markers | ACR (within 18 months: 18.1); |
|  |  |  |  |  |  |  |  | Scr/eGFR (70.0) |
|  |  |  |  |  |  |  | Anemia and malnutrition | BMI 20–25 kg/m^2^ (33.3) |
|  |  |  |  |  |  |  | Use of medications | ACEIs/ARBs (70.7); |
|  |  |  |  |  |  |  |  | Statin (50–80 years: 49.8); |
|  |  |  |  |  |  |  |  | NSAIDs (G2– G3b within 12 months: 82.6) |
|  |  |  |  |  |  |  | Management of BP | < 140/90 mmHg (54.9); |
|  |  |  |  |  |  |  |  | < 130/80 mmHg (54.0) |
| Jamaluddin 2021, Malaysia | Retrospective study | Primary care | G1- G5 | 384 | Multiple sources^c^ | Unspecified/any | **No. of themes: 6** | **No. of quality indicators: 15** |
|  |  |  |  |  |  |  | Monitoring of kidney markers | Urine protein (94.3) |
|  |  |  |  |  |  |  | CKD- MBD | Serum calcium (8.4); |
|  |  |  |  |  |  |  | (G3 or higher) | Phosphate (8.0); |
|  |  |  |  |  |  |  |  | Phosphate: (Normal: 82.6) |
|  |  |  |  |  |  |  |  | ALP (86.7) |
|  |  |  |  |  |  |  | Anemia and malnutrition | Hb (G3 or higher: 65.7) |
|  |  |  |  |  |  |  |  | Hb ≥10.0 (96.3) |
|  |  |  |  |  |  |  | Use of medications | ACEIs/ARBs (81.4); |
|  |  |  |  |  |  |  |  | Statin (92.2); |
|  |  |  |  |  |  |  |  | Avoidance of NSAIDs (89.3) |
|  |  |  |  |  |  |  | Management of BP | No specific target (45.3); |
|  |  |  |  |  |  |  |  | ≤ 140/90 mmHg (For non-DKD with <1 g/day of proteinuria: 66.7); |
|  |  |  |  |  |  |  |  | ≤ 130/80 mmHg (For DKD or non-DKD with >1 g/day of proteinuria: 37.0) |
|  |  |  |  |  |  |  | Glycemia | HbA1c (DKD 98.5); |
|  |  |  |  |  |  |  |  | HbA1c ≤ 7% (45.1) |
| Karen 2017, Canada | Cohort study | Primary care | G3 or higher (no dialysis) | 6,848 | Delphi process | Unspecified/any | **No. of themes: 5** | **No. of quality indicators: 9** |
|  |  |  |  |  |  |  | Monitoring of kidney markers | ACR (34.2); |
|  |  |  |  |  |  |  |  | Scr/eGFR (90.4) |
|  |  |  |  |  |  |  | Electrolytes | 7–30 days after the initial ACEIs/ARBs: |
|  |  |  |  |  |  |  |  | Serum potassium (59.3) |
|  |  |  |  |  |  |  | Use of medications | ACEIs/ARBs (DM and Proteinuria: 74.7); |
|  |  |  |  |  |  |  |  | ACEIs and ARBs on same day (0.7) |
|  |  |  |  |  |  |  |  | NSAIDs (1.4); |
|  |  |  |  |  |  |  |  | Stain (aged 50-80: 60.4) |
|  |  |  |  |  |  |  | Management of BP | No specific target (Proteinuria: 86.1); |
|  |  |  |  |  |  |  | (DM) | <140/90 mmHg or <130/80 mmHg (65.2) |
|  |  |  |  |  |  |  | Other | Received influenza vaccine (65.6) |
| Khanam 2019, Australia | Retrospective cohort study | General practice | G3 | 19,712 | National guidelines^a^ | Unspecified/any | **No. of themes: 3** | **No. of quality indicators: 5** |
|  |  |  |  |  |  |  |  | **(DKD vs CKD)** |
|  |  |  |  |  |  |  | Monitoring of kidney markers | ACR (68.7 vs 19.7); |
|  |  |  |  |  |  |  |  | Scr/eGFR (95.4 vs 89.6) |
|  |  |  |  |  |  |  |  | No specific target (94.1 vs 91.3) |
|  |  |  |  |  |  |  | Use of medications | LDL-C (75.4 vs 53.7); |
|  |  |  |  |  |  |  | Lipids | Total cholesterol (84.7 vs 68.1) |
| Leszek 2015^a^, Poland | Cross-sectional study | Unspecified/any | G1- G5 (no dialysis, non-transplanted) | 1,696 | Unclear sources | Hospital-based | **No. of theme: 1** | **No. of quality indicators: 4** |
|  |  |  |  |  |  |  | Use of medications | ACEIs (46.0); |
|  |  |  |  |  |  |  |  | ARBs (18.0); |
|  |  |  |  |  |  |  |  | ACEIs and ARBs (10.0); |
|  |  |  |  |  |  |  |  | ACEIs and/or ARBs (74.0) |
| Luk 2016, Asia | Cross-sectional study | Unspecified/any | unclear | 4,482 | Unclear sources | Unspecified/any | **No. of themes: 4** | **No. of quality indicators: 6 (DKD)** |
|  |  |  |  |  |  |  | Use of medications | ACEIs/ARBs (49.0); |
|  |  |  |  |  |  |  |  | Statin (53.6) |
|  |  |  |  |  |  |  | Management of BP | <130/80 mmHg (20.8) |
|  |  |  |  |  |  |  | Glycemia | HbA1c ＜7% (36.0); |
|  |  |  |  |  |  |  |  | Blood glucose (53.6) |
|  |  |  |  |  |  |  | Lipids | LDL-C < 2.6 mmol/L (49.2) |
| Manns 2017, Canada | Cohort study | Primary care | G3- G4 | 96,480 | KDIGO | Population-based | **No. of themes: 2** | **No. of quality indicators: 4** |
|  |  |  |  |  |  |  | Monitoring of kidney markers | Urine protein (Any method) (all- 82.6; 89.9 vs 78.9); |
|  |  |  |  |  |  |  | (DKD vs CKD) | Scr (73.2) |
|  |  |  |  |  |  |  | Use of medications | ACEIs/ARBs (78.3 vs 58.1); |
|  |  |  |  |  |  |  | (DKD vs CKD) | Statin (64.6 vs 39.2) |
|  |  |  |  |  |  |  |  | CKD defined by albuminuria criterion only: |
|  |  |  |  |  |  |  |  | ACEIs/ARBs (76.3 vs 26.8); |
|  |  |  |  |  |  |  |  | Statin (63.1 vs 31.6) |
| Nash 2017, Canada | Retrospective cohort study | Primary care | G3- G5 | 184,557 (aged≥40) | Delphi process | Population-based | **No. of themes: 3** | **No. of quality indicators: 7** |
|  |  |  |  |  |  |  | Monitoring of kidney markers | ACR (70.0); |
|  |  |  |  |  |  |  |  | Scr in the following 18 months (91.0) |
|  |  |  |  |  |  |  | Electrolytes | Monitoring of ACEI and ARB: |
|  |  |  |  |  |  |  | (aged≥66/ within 7 to 30 days) | Serum potassium (24.0) |
|  |  |  |  |  |  |  | Use of medications | ACEIs/ARBs (aged≥66 with CKD with ACR ≥3 mg/mmol and/or DM (75.0)); |
|  |  |  |  |  |  |  |  | ACEI and ARB (4.0); |
|  |  |  |  |  |  |  |  | Statin (aged 66 to 80 (65.0)); |
|  |  |  |  |  |  |  |  | Avoidance of NSAIDs (aged≥66 (84.0)) |
|  |  |  |  |  |  |  |  | Monitoring of ACEI and ARB (aged≥66/ within 7 to 30 days) |
|  |  |  |  |  |  |  |  | SCr (26.0) |
| Rosenthal 2006, Germany | Retrospective cohort study | Primary care | G3- G5 | 127 | K/DOQI | Population-based | **No. of themes: 8** | **No. of quality indicators: 15** |
|  |  |  |  |  |  |  | CKD- MBD | Adequately controlled (42.0); |
|  |  |  |  |  |  |  |  | Serum calcium (57.5); |
|  |  |  |  |  |  |  |  | Serum phosphorus (33.3); |
|  |  |  |  |  |  |  |  | Calcium–phosphorus product ＜ 4.44 mmol/L (39.4); |
|  |  |  |  |  |  |  |  | Parathyroid hormone (42.6) |
|  |  |  |  |  |  |  | Anemia and malnutrition | Hb (48.8) |
|  |  |  |  |  |  |  |  | Adequately controlled (80.0) |
|  |  |  |  |  |  |  |  | Serum albumin＞ 3.5 g/dl (81.9); |
|  |  |  |  |  |  |  |  | BMI＞ 20 kg/m^2^ (91.3) |
|  |  |  |  |  |  |  | Electrolytes | Serum potassium＜ 5.5 mmol/L (85.8); |
|  |  |  |  |  |  |  | Volume | Adequate control (79.5) |
|  |  |  |  |  |  |  |  | Adequate volume control (83.5) |
|  |  |  |  |  |  |  | Use of medications | ACEIs/ARBs (59.3) |
|  |  |  |  |  |  |  | Management of BP | ＜ 135/85 mmHg (39.0) |
|  |  |  |  |  |  |  | Glycemia | HbA1c (63.0 of DM) |
|  |  |  |  |  |  |  | Lipids | LDL-C＜ 2.60 mmol/L (27.6); |
| Rucker 2011, Canada | Retrospective cohort study | Unspecified/any | G3b- G5 | 31,452 | Unclear sources | Unspecified/any | **No. of themes: 3** | **No. of quality indicators: 4** |
|  |  |  |  |  |  |  | Monitoring of kidney markers | Urine protein |
|  |  |  |  |  |  |  | Use of medications | ACEIs/ARBs (with DM or significant proteinuria); |
|  |  |  |  |  |  |  | (aged ＞66) | Statin (with LDL＞ 2.5 mmol/L) |
|  |  |  |  |  |  |  | Glycemia | HbA1c (DKD) |
| Samal 2014, USA | Cross-sectional study | Primary care | G3 or G4 | 3,149 | K/DOQI | Unspecified/any | **No. of themes:3** | **No. of quality indicators: 5** |
|  |  |  |  |  |  |  | Monitoring of kidney markers | Urine protein (40.0); |
|  |  |  |  |  |  |  |  | Scr/eGFR (94.0) |
|  |  |  |  |  |  |  | Use of medications | ACEIs/ARBs (65.0) |
|  |  |  |  |  |  |  | Management of BP | <140/90 mmHg (71.0); |
|  |  |  |  |  |  |  |  | <130/80 mmHg (45.0) |
| Smits 2019, Netherlands | Retrospective cross-sectional study | Secondary care | G3a– G5 | 3,132 | Delphi process | Unspecified/any | **No. of themes: 1** | **No. of quality indicators: 3** |
|  |  |  |  |  |  |  | Use of medications | ACEIs/ARBs (56.5); |
|  |  |  |  |  |  |  |  | Statin (aged 50–65: 41.8) |
|  |  |  |  |  |  |  |  | NSAIDs (0.9) |
| Swartling 2022, Sweden | Observational cohort study | Unspecified/any | G3a- G5 | 27,847 | KDIGO | Unspecified/any | **No. of themes: 2** | **No. of quality indicators: 4** |
|  |  |  |  |  |  |  | Monitoring of kidney markers | Urine protein; |
|  |  |  |  |  |  |  |  | Scr/eGFR |
|  |  |  |  |  |  |  | Use of medications | ACEIs and/or ARBs; |
|  |  |  |  |  |  |  |  | Statin |
| Van Dipten 2017, Netherlands | Prospective observational cohort study | General practice | G3- G5 | 2,556 | National guidelines^a^ | Unspecified/any | **No. of themes: 6** | **Quality indicators: 12** |
|  |  |  |  |  |  |  | Monitoring of kidney markers | ACR (40.7); |
|  |  |  |  |  |  |  |  | Scr/eGFR (71.2) |
|  |  |  |  |  |  |  | CKD- MBD | Serum calcium (12.1); |
|  |  |  |  |  |  |  |  | Phosphate (10.1); |
|  |  |  |  |  |  |  |  | Parathyroid hormone (5.1) |
|  |  |  |  |  |  |  | Anemia and malnutrition | Hb (41.1) |
|  |  |  |  |  |  |  |  | Serum albumin (8.5) |
|  |  |  |  |  |  |  | Electrolytes | Serum potassium (58.4) |
|  |  |  |  |  |  |  | Management of BP | No specific target (64.5); |
|  |  |  |  |  |  |  |  | < 140/90 mmHg (51.2); |
|  |  |  |  |  |  |  |  | < 130/80 mmHg (20.1) |
|  |  |  |  |  |  |  | Glycemia | Fasting glucose (53.7) |
| Van Gelder 2016, Dutch | Cross-sectional study | General practice | G1- G5 | 8,794 | National guidelines^a^ | Population-based | **No. of themes: 4** | **No. of quality indicators: 10** |
|  |  |  |  |  |  |  | Monitoring of kidney markers | ACR (47.8); |
|  |  |  |  |  |  |  |  | Scr/eGFR (82.1) |
|  |  |  |  |  |  |  |  | ACEIs/ARBs (55.9); |
|  |  |  |  |  |  |  |  | Statin (47.0); |
|  |  |  |  |  |  |  |  | NSAIDs (21.3) |
|  |  |  |  |  |  |  | CKD- MBD | Vitamin D (3.7) |
|  |  |  |  |  |  |  | Management of BP | No specific target (71.9); |
|  |  |  |  |  |  |  |  | < 140/90 mmHg (43.1); |
|  |  |  |  |  |  |  |  | < 130/80 mmHg (16.4) |
|  |  |  |  |  |  |  | Glycemia | Fasting glucose (64.7) |
| Yuen 2023, Canada | Retrospective cohort study | Primary care | G3- G5 | 11,035 | Unclear sources | Population-based | **No. of themes: 4** | **No. of quality indicators: 8** |
|  |  |  |  |  |  |  | Monitoring of kidney markers | Urine protein (45.7) |
|  |  |  |  |  |  |  | Use of medications | ACEIs/ARBs (45.0; 56.0; 54.0); |
|  |  |  |  |  |  |  | (Low Risk; | Statin (33.0; 38.0; 39.0); |
|  |  |  |  |  |  |  | Intermediate Risk; | NSAIDS (10.1; 6.0; 5.0) |
|  |  |  |  |  |  |  | High Risk) | SGLT2 inhibitor (＜ 12.0) |
|  |  |  |  |  |  |  | Management of BP | No specific target (91.5; 92.0;91.0); |
|  |  |  |  |  |  |  |  | ≤ 140/90 mmHg (75.0; 67.0; 65.0) |
|  |  |  |  |  |  |  | Glycemia | HbA1c in DM (94.1; 93.0; 92.0) |

Abbreviations: CKD: chronic kidney disease; eGFR: estimated glomerular filtration rate; G: patients were identified as having CKD use eGFR criterion for CKD diagnosis; A: patients were identified as having CKD use albuminuria criterion for CKD diagnosis; ACEIs: angiotensin-converting enzyme inhibitors; ARBs: angiotensin receptor blockers; K/DOQI: Kidney Disease Outcomes Quality Initiative; CV: cardiovascular disease; NSAIDs: non-steroidal anti-inflammatory drugs; BP: blood pressure; CKD-MBD: Chronic Kidney Disease-Mineral and Bone Disorder; Scr: serum creatinine; LDL-C: low-density lipoprotein cholesterol; Hb: hemoglobin; DM: Diabetes Mellitus; DKD: Diabetic Kidney Disease; HbA1c: glycated hemoglobin; ACR: albumin-to-creatinine ratio; KDIGO: Kidney Disease Improving Global Outcomes.

a Quality indicators of five studies were derived from national guidelines, including Dutch interdisciplinary CKD-guideline (DIG-CKD), Kidney Health Australia’s CKD guidelines, Clinical Practice Guidelines (CPGs) developed by the National Healthcare Group (NHG) in Singapore, and Swedish national guidelines.

b The study by Ang 2013 reported data for multiple years; values in this table were based on the year 2011; The study by Leszek 2015 reported data for multiple years; values in this table were based on the year 2011.
c Quality indicators of two studies were based on multiple sources: one study was based on recommendations from Canadian Society of Nephrology and previously published data;13 one study used other local guidelines, the 2017 National CKD audit in the United Kingdom, a local audit, and other relevant studies.
